# Predictors of Visual Response to Intravitreal Bevacizumab for Treatment of Neovascular Age-Related Macular Degeneration

**DOI:** 10.1155/2013/676049

**Published:** 2013-08-28

**Authors:** Kai Fang, Jun Tian, Xueying Qing, Shuai Li, Jing Hou, Juan Li, Wenzhen Yu, Dafang Chen, Yonghua Hu, Xiaoxin Li

**Affiliations:** ^1^Department of Epidemiology & Biostatistics, School of Public Health, Peking University Health Science Center, Beijing 100191, China; ^2^Department of Ophthalmology, Peking University People's Hospital, Beijing 100044, China; ^3^Key Laboratory of Vision Loss & Restoration, Ministry of Education, Beijing 100044, China; ^4^Institute of Immunoprophylaxis, Beijing Centers of Disease Control & Prevention, Beijing 100013, China

## Abstract

*Purpose*. To identify the predictors of visual response to the bevacizumab treatment of neovascular age-related macular degeneration (AMD). *Design*. A cohort study within the Neovascular AMD Treatment Trial Using Bevacizumab (NATTB). *Methods*. This was a multicenter trial including 144 participants from the NATTB study. Visual outcomes measured by change in visual acuity (VA) score, proportion gaining ≥15 letters, and change in central retinal thickness (CRT) were compared among groups according to the baseline, demographic, and ocular characteristics and genotypes*. Results*. Mean change in the VA score was 9.2 ± 2.3 SD letters with a total of 46 participants (31.9%) gaining ≥15 letters. Change in median CRT was −81.5 **μ**m. Younger age, lower baseline VA score, shorter duration of neovascular AMD, and TT genotype in *rs10490924* were significantly associated with greater VA score improvement (*P* = 0.028, *P* < 0.001, *P* = 0.02, and *P* = 0.039, resp.). Lower baseline VA score and TT genotype in *rs10490924* were significantly associated with a higher likelihood of gaining ≥15 letters (*P* = 0.028, and *P* = 0.021, resp.). *Conclusions*. Baseline VA and genotype of *rs10490924* were both important predictors for visual response to bevacizumab at 6 months. This trial is registered with the Registration no. NCT01306591.

## 1. Introduction

Age-related macular degeneration (AMD) is the leading cause of blindness in people of 50 years of age or older in the developed countries [1, 2] and 80%–90% of severe vision loss and/or legal blindness can be attributed to neovascular AMD [3]. Vascular endothelial growth factor (VEGF) has been proven to play a major role in the pathogenesis of choroidal neovascularization (CNV) [4–7]. Bevacizumab (Avastin, Genentech), a monoclonal antibody to VEGF used intravenously as an anticancer agent, has been increasingly used “off-label” as an intravitreal therapy for neovascular AMD. Bevacizumab is derived from the same antibody as ranibizumab (Lucentis, Genentech) which is a smaller antigen-binding fragment and a frequently used anti-VEGF drug in the treatment of AMD [8–10]. Several studies show that bevacizumab has longer half-life in the vitreous fluid than ranibizumab because it is a full-length monoclonal antibody [11, 12], so the use of bevacizumab may reduce the frequency of visit and treatment for patients. Besides, a single dose of ranibizumab costs 40 times more than the cost of a single dose of bevacizumab [13]; this cost difference would undoubtedly have a notable influence on the patients who are treated for neovascular AMD in China. Since 2005, there have been short- and long-term retrospective and prospective studies, demonstrating the safety and efficacy of intravitreal bevacizumab for treatment of neovascular AMD [14–17]. The Neovascular Age-related Macular Degeneration Treatment Trial Using Bevacizumab (NATTB) study was the first multicenter trial designed to test the efficacy and safety of bevacizumab therapy and its validity in China. In that study, the mean increase in visual acuity (VA) measurements at 6 months was 9.20 letters compared with baseline. In spite of the improvements in VA, response to treatment seemed variable among patients. At 6 months after treatment, VA increased by ≥15 letters in 34% of the NATTB participants, while VA decreased by ≥15 letters in 3% of participants [18].

Several factors might contribute to the above variability. In the MARINA and ANCHOR studies, VA score, CNV lesion size, and age were reported to be the three most important predictors of outcome after ranibizumab treatment of neovascular AMD [19, 20]. The CATT study identified other predictors of visual outcomes after anti-VEGF treatment, such as total foveal thickness [21]. Several other studies have explored the association between genes, such as *CFH, ARMS2/HTRA1,* and *VEGF *that confer susceptibility to AMD and visual outcomes [22–24]. However, the conclusions of those studies are still inconsistent. In addition, cigarette smoking is an important environmental risk factor associated with AMD [25–27], and whether or not it also influences the response to intravitreal bevacizumab treatment must be taken into consideration. 

The present study was aimed to identify the predictors of response to bevacizumab treatment of neovascular AMD via analysis of 6-month data from the NATTB study. Demographic characteristics, behavioral factors, ocular characteristics, CNV lesion features, treatment regimens, and genotypes will be examined. To our knowledge, there have been few studies regarding the predictors of response to bevacizumab treatment of neovascular AMD in China; thus, it is necessary to elucidate the factors behind the variable response to this drug in the Chinese population. Results of this study could provide direction for evaluating the prognosis of neovascular AMD patients after bevacizumab treatment, provide basis so that patients can have appropriate expectations before receiving bevacizumab treatment, and also provide access to the mechanism of influence of patients and disease characteristics on anti-VEGF drugs.

## 2. Methods

Details of the NATTB study have been published previously [18, 24], and this study was approved by the Ethics Committee of Peking University People's Hospital. It adhered to the tenets of the Declaration of Helsinki. Each patient was fully informed of the purpose and procedures of this study, and all of them provided written informed consent before participation. This study is registered at ClinicalTrials.gov (ID no. NCT01306591). Only the main features related to evaluation of the predictors of visual outcomes are presented here. The NATTB study was a prospective, multicenter, and open-label controlled trial in which patients were randomized into 2 treatment groups each with a different regimen of administration: bevacizumab was administered every 6 weeks for a total of 8 injections (regimen A), or bevacizumab was administered every 6 weeks (3 injections) and then every 12 weeks (2 injections) (regimen B). The dose of bevacizumab was 1.25 mg (in 0.05 mL of solution). Followup of the participants was conducted at 6- or 12-week intervals for more than 6 months after the initial treatment. 

 All patients received comprehensive ophthalmologic examinations before each intravitreal injection, including measurements of the best-corrected Early Treatment Diabetic Retinopathy Study (ETDRS) visual acuity at 2 m, slit-lamp biomicroscopy, fundus examination, fundus fluorescein angiography (FFA) (Topcon TRC-50EX, Tokyo, Japan), indocyanine green angiography (ICGA) (Heidelberg Spectralis HRA, Heidelberg, Germany), and optical coherence tomography (OCT) spectral domain type, Zeiss-Humphrey, CA, USA; program, retinal mapping program version 6.2). OCT was used to measure the 1 mm central retinal thickness.

A total of 185 patients (eyes) were enrolled from January 2008 to January 2010, of which baseline behavior factors in 144 patients were available for analysis. There was no difference between the 144 patients included and the other 41 patients in terms of baseline demographics and ocular characteristics. Genotyping was also performed in the 144 patients. Genomic DNA was extracted from the peripheral blood of the patients using a DNA extraction kit (DP319-01, Tiangen Biotech, Beijing, China). The DNA samples were genotyped using the MASSARRAY Compact System (Sequenom, Inc., CA, USA). The success rate of genotyping was 98%.

 Predictors of 3 visual response measures at the 6th month were evaluated, including change in VA score from baseline, proportion of patients that gained ≥15 letters from baseline, and change in central retinal thickness (CRT) from baseline. For the exploratory association analysis of the NATTB data, factors were considered including patients' baseline age, gender, cigarette smoking status, VA score, CNV lesion type, duration of neovascular AMD (defined as the interval from diagnosis of neovascular AMD to participation in the study), treatment regimen, and genotype. 

The values for the change in VA scores are presented as mean ± standard deviation (SD). Variables of manifold classification were evaluated by one-way analysis of variance, using generalized linear models when the variance between groups was homogeneous; otherwise, nonparametric testing (the Kruskal-Wallis *H* Test) was used. Variables of dichotomy classification were evaluated by the unpaired *t*-test. Variables with a *P* < 0.05 in the univariate model, or reported by previous trials, were included in a multivariate linear regression model to evaluate the independent effects of these predictors using a backward selection procedure. The proportion of ≥15 letters gain for each predictor was evaluated by the *χ* ^2^ test, and the multivariate analysis was performed in a logistic regression model using a backward selection procedure. The distribution of the change in CRT was asymmetrical, so the values are presented as median, and each predictor was evaluated by nonparametric testing (the Kruskal-Wallis *H* Test or the Mann-Whitney *U* test). *P* values < 0.05 were considered statistically significant. Dunnett's *t*-test or the Bonferroni methods were used for multiple comparisons. All data analyses were performed using SPSS (version 16.0 for windows; SPSS, Inc., IL, USA).

## 3. Results

### 3.1. Demographic and Eye Characteristics of the Study Participants

The demographic and eye characteristics of the 144 participants are shown in Table 1. There were 74 participants in regimen A and 70 participants in regimen B. The demographic characteristics, baseline eye characteristics, and visual outcomes at the 6th month were balanced in the two regimens. Overall, the mean age was 68.8 ± 8.6 years: 66.0% were men and 46.2% were former or current cigarette smokers. The mean baseline and the 6-month VA scores were 37.5 ± 18.4 letters and 46.7 ± 20.2 letters, respectively. A total of 44.9% of CNV lesions were occult only, 16.5% were minimally classic CNV, and 38.6% were predominantly classic CNV. The duration of neovascular AMD of nearly half of the participants was 1 to 6.9 months. The medians of the baseline and the 6-month CRT were 344.5 *μ*m and 229.0 *μ*m, respectively.

### 3.2. Predictors of VA Score Change (Letters) from Baseline at 6 Months

**Figure 1:**
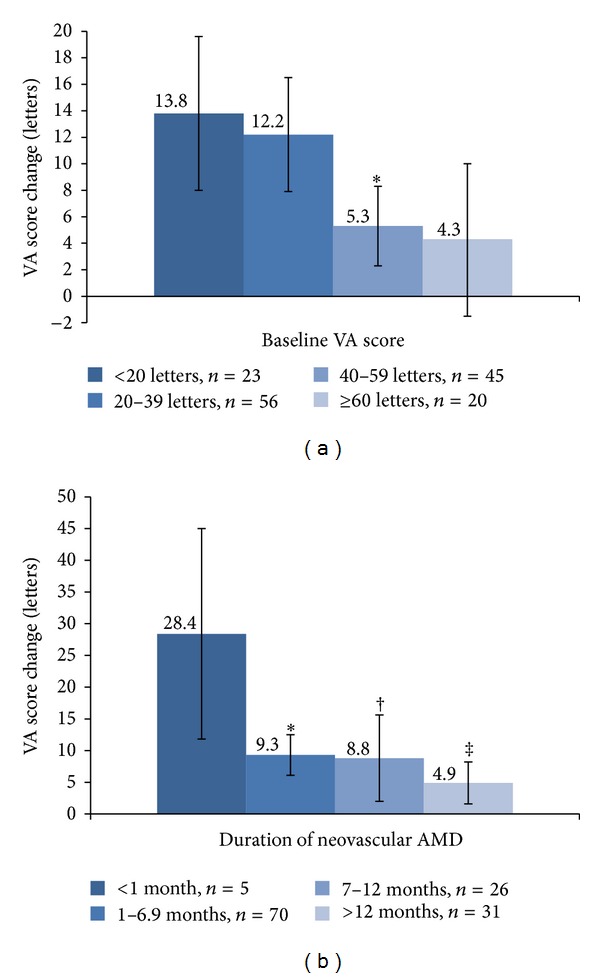
(a) Association of baseline visual acuity (VA) score with VA score change at 6 months. Baseline VA score was significantly associated with VA score change at 6 months (*P* = 0.015). *P = 0.036 (the first group was the reference). (b) Association of duration of neovascular age-related macular degeneration (AMD) with VA score change at 6 months. Duration of neovascular AMD was significantly associated with VA score change at 6 months (*P* = 0.005). *P = 0.005; ^†^ *P* = 0.007; ^‡^ *P* = 0.001 (the first group was the reference).

The mean change of VA score from baseline at the 6th month was 9.2 ± 2.3 letters. In the univariate analysis, the baseline VA score (Figure 1(a), *P* = 0.015) and the duration of neovascular AMD (Figure 1(b), *P* = 0.005) were significantly associated with a VA score change at 6 months. Compared with regimen B, patients in regimen A had a better change in VA score at 6 months (*P* = 0.035) (Table 2). There was no significant association found between age, gender, cigarette smoking status, and CNV lesion type and the VA score change from baseline at the 6th month (Table 2).

The association between the VA score change (letters) from baseline at the 6th month and SNPs (*CFH rs800292, ARMS2 rs10490924, *and* HTRA1 rs11200638*) is shown in Table 3. The TT genotype of *rs10490924* and the AA genotype of *rs11200638* had the worst VA score changes (*P* = 0.005, and *P* = 0.002, resp.). *CFH rs800292* was not significantly associated with VA score change (*P* = 0.065).

Finally, predictors of VA score change (letters) were analyzed in a multivariate model; the results are shown in Table 4. Age (*P* = 0.028), baseline VA score (*P* < 0.001), duration of neovascular AMD (*P* = 0.02), and *rs10490924* genotype (*P* = 0.039) were retained in the final model. Since *rs10490924* and *rs11200638* were in high linkage disequilibrium, only one SNP was included in the multivariate model. Baseline VA score had the greatest influence on the VA score change from baseline to the 6th month.

### 3.3. Predictors of a ≥15-Letter Gain from Baseline at 6 Months

**Figure 2:**
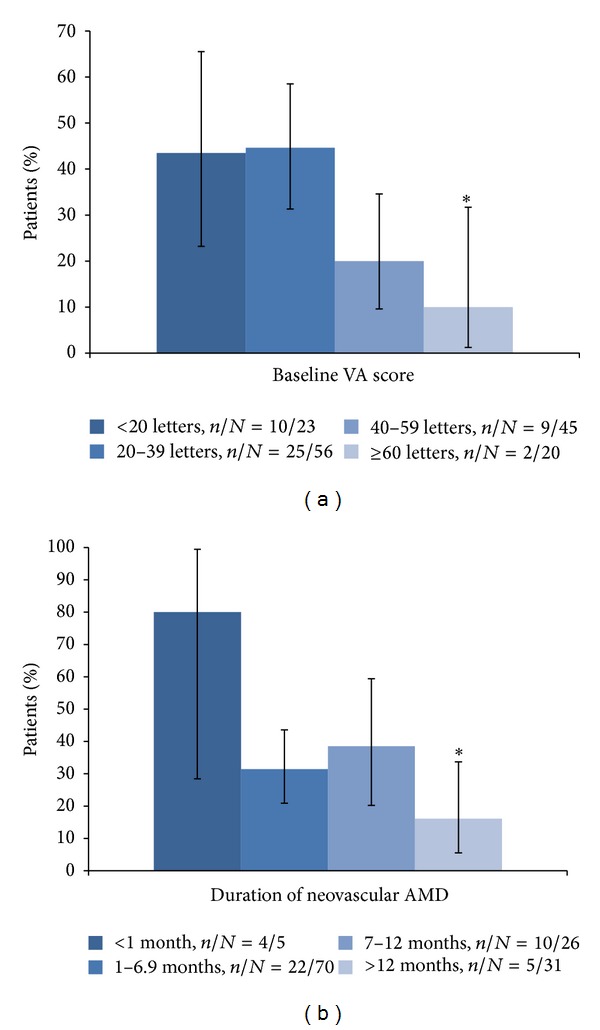
(a) Association of baseline visual acuity (VA) score with proportion of ≥15 letters gaining from baseline at 6 months. Baseline VA score was significantly associated with proportion of ≥15 letters gaining from baseline at 6 months (*P* = 0.005). *P = 0.015 (the first group was the reference). (b) Association of duration of neovascular age-related macular degeneration (AMD) with proportion of ≥15 letters gaining from baseline at 6 months. Duration of neovascular AMD was significantly associated with proportion of ≥15 letters gaining from baseline at 6 months (*P* = 0.021). *P = 0.009 (the first group was the reference).

There were 46 participants (31.9%) who gained ≥15 letters in VA score from baseline to the 6th month. The univariate results for the baseline characteristics of the patients that gained ≥15 letters at the 6th month are shown in Table 2. The baseline VA score (Figure 2(a), *P* = 0.005) and the duration of neovascular AMD (Figure 2(b), *P* = 0.021) were significantly associated with the gain. 

The results of the analysis of the association with SNPs are shown in Table 3. *CFH rs800292, ARMS2 rs10490924,* and *HTRA1 rs11200638* were all found to be associated with the gain of ≥15 letters (*P* = 0.041, *P* = 0.015, and *P* = 0.027, resp.).

For the multivariate logistic regression analysis (Table 5), baseline VA score (*P* = 0.028), duration of neovascular AMD (*P* = 0.092), and *rs10490924* genotype (*P* = 0.021) were retained in the final model. Compared with the patients whose baseline VA was less than 20 letters, the *OR *(*95% CI*) for gaining ≥15 letters was 0.277 (0.081, 0.944) in patients with a baseline VA of 40 to 59 letters, and it was 0.107 (0.018, 0.638) in patients with a baseline VA of more than 60 letters.

### 3.4. Predictors of Central Retina Thickness Change from Baseline at 6 Months

The median change in CRT from baseline to the 6th month was −81.5 *μ*m. The univariate results for the baseline characteristics of the CRT change from baseline to the 6th month are shown in Table 2. We obtained the measure of CRT change in 132 of the 144 patients. There was no significant association between any of the baseline characteristics and change in CRT at the 6th month, with the exception of cigarette smoking. The median change in CRT in those who had never smoked was greater than the median CRT change in the sformer or current cigarette smokers (−106.5 *μ*m versus −56.0 *μ*m; *P* = 0.047).

None of the 3 SNPs showed any association with CRT change (Table 3).

## 4. Discussion

In the present study, age, baseline VA score, duration of neovascular AMD, and *ARMS2/HTRA1* genotypehave been identified as the predictors of the visual response to bevacizumab treatment at 6 months. Baseline VA score and *ARMS2/HTRA1* genotype were both associated with the two measurements of visual response, the VA score change (letters), and the proportion of those gaining ≥15 letters. Cigarette smoking was found to be associated with an OCT feature: central retinal thickness (CRT) change.

The MARINA and ANCHOR studies have both shown that baseline VA was the most important predictor of VA outcome with ranibizumab treatment [19, 20]. Similar to those studies, we also identified baseline VA as the most influential predictor of VA outcome with bevacizumab treatment. Consistent with the CATT study, we found that the worse the baseline VA of the eyes, the more significant improvement in VA [21]. One explanation may be that in the patients with the best baseline VA vision cannot be completely restored to what it had been before the presence of CNV, so their change in VA may be less than that of the patients with a worse baseline VA. This idea is supported by the inverse correlation, found in the current study, between the baseline VA score and the proportion of those with ≥15 letters gained. The association between baseline age and VA score change found in our study is also consistent with previous findings [19–21]. According to the multivariate analysis, the change in baseline VA decreased as age increased. We found a decrease in the change from baseline VA of ~3 letters for every 10 years of age. However, the negative results of age with the proportion of those gaining ≥15 letters may have resulted from information loss after transforming a continuous variable into a categorical variable. Moreover, 6 months is not a long duration; thus, it is possible that the outcome measurement of ≥15 letters gained will have a higher statistical efficiency in longer-term data analysis.

The duration of neovascular AMD is a new predictor that was identified in the present study. However, two previous studies failed to confirm any association between the duration of neovascular AMD and the VA outcomes [19, 20]. Different inclusion criteria may be one reason for the difference. Compared with the previous studies, the eligible baseline VA criteria of the NATTB study were broader (between 5 letters and 73 letters), so more patients were considered to have experienced a longer duration of AMD in this study. In other words, the different composition of participants may have led to the different results. This finding implies that patients should receive appropriate treatment once diagnosed with a CNV.

The relationship between *ARMS2/HTRA1* and VA score change from baseline to the 6th month is consistent with the previous analysis of visual outcome measures at 3 months in the NATTB study [24]. Furthermore, the effect of the genes on the visual outcomes was independent of other predictors. We revealed that eyes with the TT genotype in *ARMS2 rs10490924* or with the AA genotypes in *HTRA1 rs11200638* had a smaller improvement in VA. Several previous studies did not find the association between the response to anti-VEGF therapy (ranibizumab or bevacizumab) and the genotype in *ARMS2 rs10490924 *[20, 28–30], however; 2 other studies have reported that *HTRA1* may influence the response to treatment of neovascular AMD with ranibizumab [31, 32]. As one of the most important susceptibility genes for AMD, the mechanism of how the *ARMS2/HTRA1* genes influence the occurrence and development of AMD has been widely studied, and their association with the response to anti-VEGF treatment is still controversial. More research is needed on this mechanism in order to elucidate the impact of the *ARMS2/HTRA1* genes. The association of the *CFH rs800292* genotype with the ≥15 letters gain from baseline at 6 months was significant. However, it was found to be negative in the multivariate model as this genotype did not appear to be associated with a change in VA score from baseline to the 6th month; this finding was inconsistent with the results of the 3-month data analysis [24]. More short- and long-term observations on the association between* CFH rs800290* and the response to bevacizumab treatment are needed to resolve this inconsistency.

Cigarette smoking, which has been identified as the strongest environmental risk factor for the development of AMD, also showed an association with the change in CRT from baseline to the 6th month. *In vitro* and *in vivo*, nicotine has been found to upregulate the expression of VEGF and was reported to be responsible for the increase in the VEDF/PEDF ratio in RPE cells [33, 34], and oxidative injury induced by hydroquinone, a major prooxidant in cigarette smoke might lead to increased expression of VEGF protein and decreased expression of PEDF protein [35]. Furthermore, dioxin which is present primarily in the gaseous phase of cigarette smoking promotes VEGF production in the retina of mice and human retinal pigment epithelium (RPE) cells and exacerbates the development of laser-induced CNV [36]. Cigarette smoking plays a role in the pathogenesis of neovascular AMD in more than one way, including causing oxidative damage [37, 38], as well as affecting choroidal blood flow [39, 40] and macular pigment optical density [41, 42]; therefore, smoking may have an antieffect of bevacizumab treatment on the pathological characteristics in the retina for neovascular AMD, and, as a distant factor for retina, smoking may cause the anatomical change at first and then lead to dysfunction of retina, which may explain why cigarette smoking showed no association with VA score change that was a measurement of visual function.

Limitations of this study include that people in the cohort were not treated equally with bevacizumab and its small sample size. Although it was seen that there was significant difference in letters gained between treatment group A and group B in the 6th month data analysis, the patients in group A were actually treated only one more time compared with the patients in group B. Moreover, treatment regimen variable was not retained in the multivariate model, so the difference between the two groups in the 6th month data might result from other important predictors such as baseline VA score. The results were even similar in the two groups after association analyses between demographic and eye characteristics and VA score change from baseline at 6 months were conducted in the two regimen groups, respectively. So, the data of the two groups were combined to be analyzed in consideration of power issue. And further study might compare factors influencing the visual outcomes between the two groups if increasing the cohort size.

In summary, based on the analysis of the 6-month data from the NATTB study, we found that age, baseline VA score, duration of neovascular AMD, and *ARMS2/HTRA1 *genotype were all independent predictors of VA score change. Of these, baseline VA score was the most important predictor of visual response at 6 months following bevacizumab treatment of neovascular AMD. Cigarette smoking was found to decrease the improvement of CRT. Analysis of subsequent follow-up data may reveal a long-term effect of anti-VEGF treatment at different levels of these predictors.

## Figures and Tables

**Table 1:** Demographic and ocular characteristics of 144 patients in two regimens.

Characteristics	Overall (*n* = 144)	Regimen A (*n* = 74)	Regimen B (*n* = 70)	*P*
Age (years)				
Mean (SD)	68.8 (8.6)	67.7 (9.1)	70.0 (8.0)	0.111
Range				
Age group *n* (%)				
50–59 years	26 (18.1)	17 (23.0)	9 (12.9)	0.231
60–69 years	43 (29.9)	22 (29.7)	21 (30.0)	
70–79 years	62 (43.1)	31 (41.9)	31 (44.3)	
≥80 years	13 ( 9.0)	4 (5.4)	9 (12.9)	
Gender *n* (%)				
Male	95 (66.0)	47 (63.5)	48 (68.6)	0.522
Female	49 (34.0)	27 (36.5)	22 (31.4)	
Smoking* *n* (%)				
No	77 (53.8)	40 (54.8)	37 (52.9)	0.816
Yes	66 (46.2)	33 (45.2)	33 (47.1)	
VA score (letters) Mean ± SD				
Baseline	37.5 ± 18.4	35.1 ± 18.5	40.1 ± 18.1	0.099
6 months	46.7 ± 20.2	46.6 ± 20.0	46.8 ± 20.5	0.954
Lesion type* *n* (%)				
Occult only	57 (44.9)	27 (42.9)	30 (46.9)	0.467
Minimally classic	21 (16.5)	13 (20.6)	8 (12.5)	
Predominantly classic	49 (38.6)	23 (36.5)	26 (40.6)	
Duration of neovascular AMD* *n* (%)				
<1 month	5 (3.8)	0 (0.0)	5 (7.6)	0.067
1–6.9 months	70 (53.0)	40 (60.6)	30 (45.5)	
7–12 months	26 (19.7)	11 (16.7)	15 (22.7)	
>12 months	31 (23.5)	15 (22.7)	16 (24.2)	
CRT (*μ*m)* median				
Baseline	344.5	349.0	344.5	0.346
6 months	229.0	227.5	230.0	0.667

AMD: age-related macular degeneration; CRT: central retinal thickness; SD: standard deviation; VA: visual acuity.

*Variable that had missing values.

**Table 2:** Predictors of visual acuity score change and central retinal thickness change from baseline at 6 months.

Baseline characteristics	VA score change					CRT change (*μ*m)		
	Letters			≥15 letters gain		*N*	Median	P*
	*N*	Mean ± SD	*P*	*n* (%)	*P*			
Age (years)								
50–59	26	13.4 ± 10.9	0.227	12 (46.2)	0.393	22	−47.0	0.504
60–69	43	9.9 ± 15.1		13 (30.2)		42	−130.0	
70–79	62	6.8 ± 14.2		17 (27.4)		58	−77.5	
≥80	13	10.0 ± 12.2		4 (30.8)		10	−128.5	
Gender								
Male	95	9.1 ± 14.7	0.928	31 (32.6)	0.805	86	−64.0	0.052
Female	49	9.3 ± 12.2		15 (30.6)		46	−122.0	
Smoking								
No	77	9.3 ± 14.3	0.996	26 (33.8)	0.659	72	−106.5	0.047
Yes	66	9.3 ± 13.5		20 (30.3)		59	−56.0	
Lesion type								
Occult only	57	7.8 ± 10.6	0.832*	13 (22.8)	0.104	55	−67.0	0.352
Minimally classic	21	11.1 ± 18.8		10 (47.6)		20	−117.0	
Predominantly classic	49	8.4 ± 15.2		15 (30.6)		47	−82.0	
Treatment regimen			0.035		0.626			0.492
A	74	11.6 ± 12.6		25 (33.8)		65	−103.0	
B	70	6.7 ± 14.8		21 (30.0)		67	−56.0	

CRT: central retinal thickness; SD: standard deviation; VA: visual acuity.

*Nonparametric testing.

**Table 3:** Association between single-nucleotide polymorphisms and changes in visual acuity score and central retinal thickness.

SNP	VA score change					CRT change (*μ*m)		
	Letters			≥15 letters gain		*N*	Median	P*
	*N*	Mean ± SD	*P*	*n* (%)	*P*			
*CFH rs800292 *								
TT	11	17.6 ± 17.6	0.065	7 (63.6)	0.041	11	−120.0	0.620
TC	56	10.0 ± 14.0		19 (33.9)		53	−47.0	
CC	73	7.3 ± 13.1		19 (26.0)		65	−71.0	
*ARMS2 rs10490924 *								
GG	16	12.4 ± 10.9	0.005	6 (37.5)	0.015	16	−134.0	0.445
GT	44	14.0 ± 13.5		21 (47.7)		38	−69.0	
TT	83	6.0 ± 13.9		19 (22.9)		77	−81.0	
*HTRA1 rs11200638 *								
GG	16	12.4 ± 10.9	0.002	6 (37.5)	0.027	16	−134.0	0.473
GA	43	14.5 ± 12.7		20 (46.5)		37	−71.0	
AA	85	5.9 ± 14.1		20 (23.5)		79	−81.0	

CRT: central retinal thickness; SD: standard deviation; SNP: single-nucleotide polymorphism; VA: visual acuity.

*Nonparametric testing.

**Table 4:** Multivariate analysis of visual acuity score change (letters) from baseline at 6 months.

Predictors	Unstandardized coefficients *B* (SE)	Standardized coefficients *B*	*t*	*P*
Age	−2.998 (1.347)	−0.188	−2.227	0.028
Baseline VA score	−4.561 (1.217)	−0.303	−3.749	<0.001
Duration of neovascular AMD	−3.040 (1.290)	−0.193	−2.357	0.020
*ARMS2 rs10490924 *	−3.593 (1.720)	−0.178	−2.090	0.039

AMD: age-related macular degeneration; SE: standard error; VA: visual acuity.

Variables included in step 1 are age group, gender, baseline VA score, duration of neovascular AMD, *ARMS2 rs10490924* genotype, and treatment regimen.

**Table 5:** Multivariate analysis of ≥15 letters gain from baseline at 6 months.

Predictors	*N*	*n* (%)	OR (95% CI)	*P*
Baseline VA score				
<20 letters	23	10 (43.5)	1.000	0.028
20–39 letters	56	25 (44.6)	0.688 (0.227–2.091)	
40–59 letters	45	9 (20.0)	0.277 (0.081–0.944)	
≥60 letters	20	2 (10.0)	0.107 (0.018–0.638)	
Duration of neovascular AMD				
<1 month	5	4 (80.0)	1.000	0.092
1–6.9 months	70	22 (31.4)	0.105 (0.010–1.113)	
7–12 months	26	10 (38.5)	0.134 (0.012–1.542)	
>12 months	31	5 (16.1)	0.047 (0.004–0.571)	
*ARMS2 rs10490924 *				
GG	16	6 (37.5)	0.742 (0.190–2.897)	0.021
GT	44	21 (47.7)	1.000	
TT	83	19 (22.9)	0.284 (0.114–0.706)	

AMD: age-related macular degeneration; CI: confidence interval; OR: odds ratio; VA: visual acuity.

Variables included in step 1 are age group, gender, baseline VA score, duration of neovascular AMD, *ARMS2 rs10490924* genotype, and treatment regimen.
